# Characterizing dynamic local functional connectivity in the human brain

**DOI:** 10.1038/srep26976

**Published:** 2016-05-27

**Authors:** Lifu Deng, Junfeng Sun, Lin Cheng, Shanbao Tong

**Affiliations:** 1 School of Biomedical Engineering, Shanghai Jiao Tong University, Shanghai, 200030, China

## Abstract

Functional connectivity (FC), obtained from functional magnetic resonance imaging (fMRI), brings insights into the functional organization of the brain. Recently, rich and complex behaviour of brain has been revealed by the dynamic fluctuation of FC, which had previously been regarded as confounding ‘noise’. While the dynamics of long-distance, inter-regional FC has been extensively studied, the dynamics of local FC within a few millimetres in space remains largely unexplored. In this study, the local FC was depicted by regional homogeneity (ReHo), and the dynamics of local FC was obtained using sliding windows method. We observed a robust positive correlation between ReHo and its temporal variability, which was shown to be an intrinsic feature of the brain rather than a pure stochastic effect. Furthermore, fluctuation of ReHo was associated with global functional organization: (i) brain regions with higher centrality of inter-regional FC tended to possess higher ReHo variability; (ii) coherence of ReHo fluctuation was higher within brain’s functional modules. Finally, we observed alteration of ReHo variability during a motor task compared with resting-state. Our findings associated the temporal fluctuation of ReHo with brain function, opening up the possibility of dynamic local FC study in the future.

In the past two decades, the bloom of resting-state functional magnetic resonance imaging (rs-fMRI) has greatly expanded our understanding of the human brain. As human brain organizes as a complicated network in terms of structure and function, rs-fMRI studies primarily focus on functional connectivity (FC) between brain regions^1^. Until recent years, most studies of rs-fMRI were conducted under the assumption that the FC in human brain remains stationary in resting state, and longer scanning time was recommended to improve the reliability of the results. However, recent studies have shown that dynamic changes in FC could be observed even within a single scan^2^,^3^,^4^. What’s more, the behaviour of dynamic FC were found to be associated with cognitive states^5^,^6^,^7^, and the dynamics also alters in various neuropathological or psychiatric situations, such as Alzheimer’s disease^8^, schizophrenia^9^, and epilepsy^10^,^11^. New tools and methods have been introduced for analysing the dynamic FC^6^,^11^,^12^. Some controversies, such as distinguishing the true dynamic FC under the ambient statistical noise^12^,^13^, have also been examined. In short, dynamic FC, being a blooming area in neuroscience, has continuously brought insights into the human brain.

Most FC studies focused on global connectivity between spatially separated brain regions, ignoring local functional interaction between spatially adjacent regions. To quantify local FC in human brain, several measures were proposed, including the most commonly used regional homogeneity^14^ (ReHo), as well as integrated local correlation^15^ (ILC), and local functional connectivity density (lFCD). Insights into the role of localized brain functionality in larger brain networks have been revealed by ReHo and other local connectivity measures. The activity of default-mode network (DMN) in resting state as observed by ReHo, was found to be consistent with observations using FC and independent component analysis^16^ (ICA). While the functional hub in the posterior cingulate and ventral precuneus was depicted by lFCD^17^, ReHo in the ventral visual stream was discovered to be correlated with the hierarchy of information processing^18^. In addition, ReHo alteration has been associated with diseases such as schizophrenia^19^, depression^20^,^21^, Alzheimer’s disease^22^, and autism spectrum disorder^23^. Other studies have also reported relationships between ReHo and many factors including age^24^, gender^24^,^25^, and intelligence^26^. These findings indicated that the localized brain connectivity is associated with the physiological, pathological, and psychological changes in human brain^27^.

Despite the recent developments in dynamic global FC analysis, knowledge regarding the dynamic behaviours of regional FC are still rather limited. Presently, the temporal variability of ReHo is generally considered as troublesome nuisance that should be eliminated in order to improve the reliability or repeatability of results. Nevertheless, in 2014, Hudetz *et al.* reported reduction of ReHo variability in rats undergoing deep anaesthesia, compared with rats in light anaesthesia^28^, suggesting a linkage between brain function and ReHo variability. However, the role that such a linkage would play is far from being understood, and the temporal variability of ReHo in human brain remains largely unexplored. Does the dynamics of ReHo reflect the underlying brain state? Is the variability of ReHo in different brain regions associated with the functional roles of those regions? Besides anaesthesia, will the alteration of variability in local connectivity be associated with other physiological or behavioural changes? These questions remain open to investigation.

The primary focus of this research is to characterize the temporal fluctuation of local FC. As the starting point of our analysis, dynamic ReHo series was measured by calculating the ReHo inside a short time window that ‘slides’ from the beginning of the data till the end. Two fMRI datasets were investigated: one was from the Human Connectome Project and the other was collected from normal subjects at rest and during performing hand-closing-opening (HCO) task. With the first dataset, we first examined the relationship between ReHo and ReHo variability (ReHoV) comprehensively (To avoid ambiguity, in the following text, ‘ReHo’ refers to the result calculated from the entire scan, unless specifically noted). We then asked whether the ReHo-ReHoV relationship we observed is intrinsic or not, and attempted to answer this question by performing surrogate test with randomized data and the real data. Furthermore, for each brain region of interest (ROI), we explored the relationship between its ReHoV and its centrality in the global functional network of the brain. The coherence of ReHo fluctuation across different ROIs was also explored, and the organization of such coherence was characterized with respect to the functional modules of the brain. Additionally, with the HCO task data, we investigated whether ReHoV was modulated by a motor-related task. This study enriches our understanding of regional brain connectivity, and underscore the need for further exploration of the brain’s dynamic FC at a local scale.

## Results

### The Correlation between ReHo and ReHo Variability

**Figure 1:**
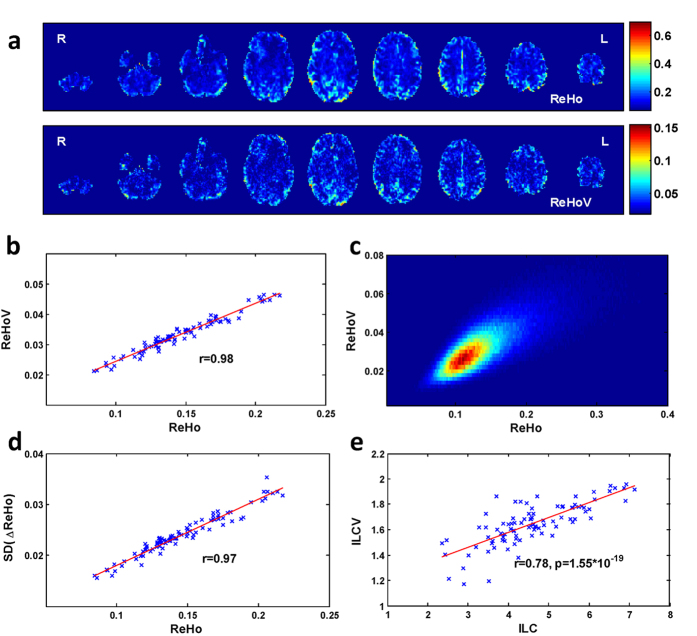
The correlation between ReHo and ReHoV from an exemplar subject. **(a)** cross-sectional view of ReHo (top panel) and ReHoV (below panel); **(b)** scatter plot of ROI-wise mean ReHo and mean ReHoV in ROIs defined by Automated Anatomical Labelling (AAL) template (see Materials and Methods), each cross represents an ROI; **(c)** joint probability distribution of ReHo and ReHoV across all voxels in grey matter (defined as the total volume of all the AAL ROIs); **(d)** ROI-wise mean variability of ReHo, measured as the standard deviation of first order difference of sliding-window ReHo series (i.e. *SD*(Δ*ReHo*)) with respect to the mean ReHo in ROIs defined by AAL; **(e)** the corresponding local connectivity and its variability measured by ILC algorithm in ROIs defined by AAL.

We observed similar spatial pattern between ReHo and ReHoV for each subject (Fig. 1a). To quantify the similarity between spatial patterns of ReHo and ReHoV, we performed correlation analysis between the mean ReHo and mean ReHoV across ROIs for each subject, and found a significant linear trend that ROIs with higher ReHo also have higher ReHoV (i.e., higher variance of ReHo across sliding windows) (Fig. 1b). Such correlation also appeared in an alternative ROI definition (Supplementary Fig. S1a). The ReHo-ReHoV correlation appeared in all subjects (Supplementary Table S2). Furthermore, ReHo and ReHoV also showed apparent correlation across voxels, as demonstrated in their joint probabilistic distribution function (PDF) (Fig. 1c). These results suggested that the linear correlation between ReHo and ReHoV is not a coincidence caused by ROI definition.

The correlation between ReHo and ReHoV was robust to measurements and parameters. The standard deviation of ReHo difference between two successive windows, as an alternative measure of ReHo fluctuation, also strongly correlated with ReHo (Fig. 1d). Strong linear correlation between ReHo and ReHoV always existed under different sliding windows and between-window overlaps, (Supplementary Fig. S1b). Besides, ILC, as an alternative measure of local connectivity, significantly correlated with ILC variability (ILCV) (Fig. 1e), indicating that the correlation between local FC and its variability is measure-independent.

**Figure 2:**
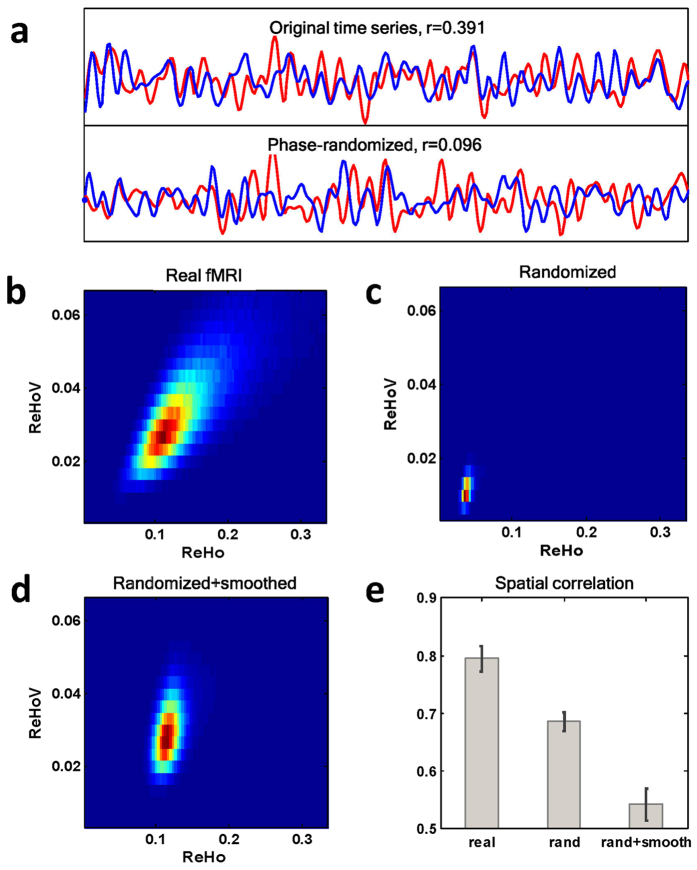
Surrogate test of the correlation between ReHo and ReHoV. **(a)** Example of the original fMRI time series (above) from two adjacent voxels, and their surrogate data (below); **(b–d)** joint PDFs between ReHo and ReHoV across all voxels of one exemplar subject from the real fMRI data, the phase-randomized data, and the smoothed phase-randomized data, respectively; **(e)** the spatial correlation between ReHo and ReHoV in the original fMRI data, their phase-randomized surrogate data, and their smoothed phase-randomized surrogate data, respectively. Error bars indicate variances across subjects.

The high correlation between ReHo and ReHoV could possibly be a result of stochastic process related to the property of fMRI image, blood-oxygenation-level dependent (BOLD) signal, or the ReHo algorithm itself. To explore these possibilities, we used surrogate data to test the ReHo-ReHoV correlation in a ‘non-functioning’ brain. As a demonstration of the surrogate data generation, we randomly selected a pair of adjacent voxels in one subject, then assigned random phases to the time courses of both voxels in the frequency domain independently, making the two time courses desynchronized (Fig. 2a). For the data from Human Connectome Project, the fMRI data of each subject were fully randomized (i.e., all voxels were phase-randomized). Besides, since intrinsic spatial smoothness of the image may increase the ReHo value, a smoothed version of surrogate data with the smoothness similar to the original data was also generated for following analysis (see Materials and Methods for detail).

As expected, phase-randomized data displayed low ReHo and low ReHoV, in contrast to the higher level of ReHo and ReHoV in the smoothed randomized data, though none of them exceed those for real data (Fig. 2b–d). The joint PDFs showed the ReHo-ReHoV dependency in either surrogate data was weaker than that in real fMRI data. The analysis on spatial similarity between ReHo and ReHoV revealed that, the real fMRI data possessed intrinsic correlation between ReHo and ReHoV, and this correlation was substantially weaker in surrogate data, especially in smoothed phase-randomized data (Fig. 2e).

### Towards Functional Characterization of ReHo Fluctuation

**Figure 3:**
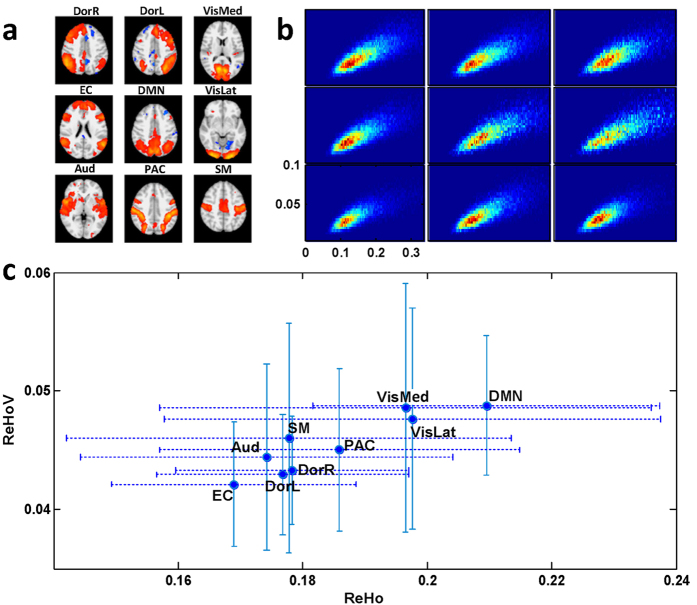
**(a)** The nine ICNs of interest obtained by ICA; **(b)** ReHo-ReHoV joint PDFs in the nine ICNs of a typical subject; **(c)** ReHo and ReHoV in each ICN averaged across all subjects. Error bars indicate variances across subjects.

We further investigated the distribution of ReHoV in different functional modules. These functional modules were referred to as independent component networks (ICNs) in the brain, obtained by independent component analysis (ICA) (see Materials and Methods for detail). Nine ICNs of interest were identified for analysis (Fig. 3a), namely the default-mode network (DMN), medial visual network (VisMed), lateral visual network (VisLat), left and right dorsal visual streams (DorL and DorR), executive control network (EC), auditory network (Aud), sensory-motor network (SM), and parietal association cortex (PAC), as described in literature^29^,^30^.

Using the binarized spatial probability maps of the 9 ICNs as masks, we observed the ReHo-ReHoV correlation in the joint PDFs in each ICN (Fig. 3b). Noted that ReHo and ReHoV varied in different ICNs. Figure 3c showed the mean ReHo vs mean ReHoV in each ICN across subjects, indicating distinct ReHo and ReHoV levels in different functional networks, with DMN and the two visual networks possessing the highest ReHoV and ReHo. While there is a trend that the ICN with higher ReHo is associated with higher ReHoV, the remaining variance in group mean ICN-wise ReHo-ReHoV correlation in Fig. 3c indicated that ReHo and ReHoV were non-equivalent.

**Figure 4:**
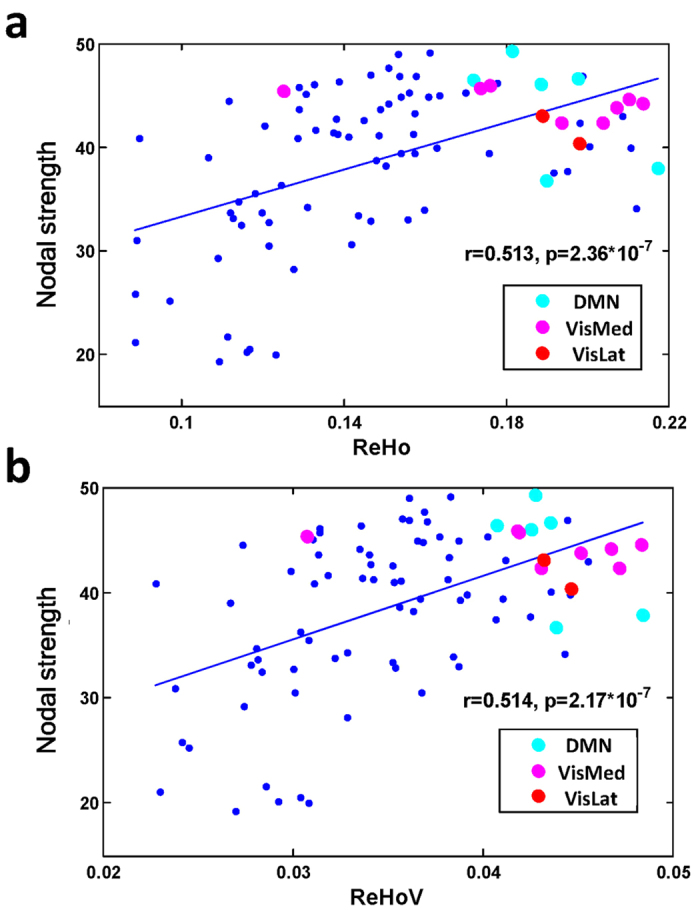
**(a)** The positive correlation between nodal strength and group averaged ReHo, and **(b)**the positive correlation between nodal strength and group averaged ReHoV. Each dot represents one ROI defined by AAL template. Cyan, magenta, and red dots highlight ROIs that overlap most with DMN, medial visual network (VisMed), and lateral visual network (VisLat), respectively, which have the highest ReHo and ReHoV.

In brain networks, the regions widely connecting with other brain regions are regarded as ‘hubs’. We analysed group-averaged ReHo mapping and group-averaged functional network, and showed that both ReHo and ReHoV positively correlated with nodal strength of the regions (Pearson’s correlation, N = 90, Fig. 4a,b), indicating that the ‘hub’ nodes (e.g., post cingulate and precuneus inside DMN) tended to have high ReHo and high ReHoV. Such trends were also prevalent on individual level (Supplementary Table S3).

**Figure 5:**
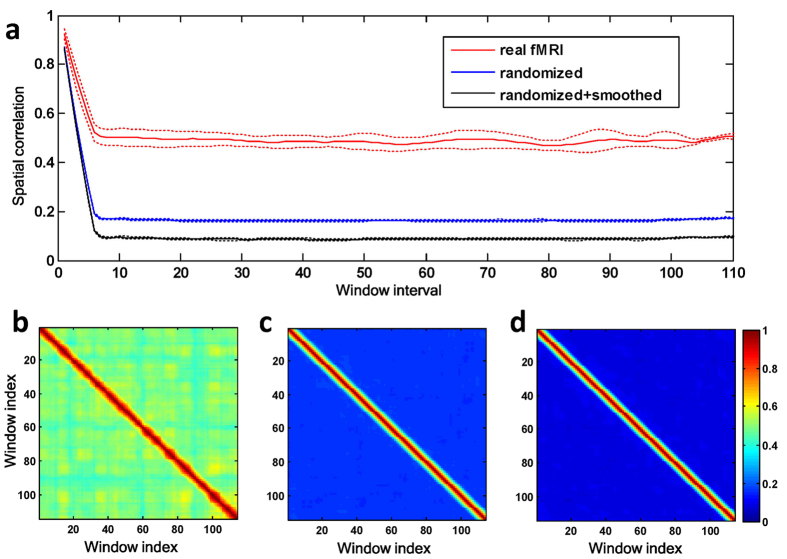
Temporal characteristics of ReHo fluctuation from a typical subject. **(a)** The spatial correlation between ReHo maps of two windows as a function of the window interval. Here, the window interval was defined as the absolute difference between the indexes of the two windows. The spatial correlation is averaged across all pairs of windows that have the same interval. Dashed lines indicate the standard deviation across window-pairs having the same intervals. Similarity matrices showed the spatial correlation between ReHo maps of any pair of windows in real fMRI data **(b)**, randomized data **(c)**, and randomized + smoothed data **(d)**, respectively.

To quantify the temporal fluctuation of ReHo from window to window, we measured the spatial correlation between whole-brain ReHo of two different windows with respect to the time interval in between (Fig. 5a, windows length: 60 TRs or 43.2 sec; window overlap: 10 TRs or 7.2 sec). The average spatial correlation in all cases decreased with the increase of interval till there was no overlap in between (i.e., 43.2 sec). For real fMRI data, the spatial correlation maintained at a medium level when two windows were distant in time, while for the surrogate data, spatial correlation disappeared once two windows no longer overlapped. Moreover, the variance of the spatial correlation across all possible combinations of window-pairs of equal time interval was much higher in real fMRI data than in their corresponding surrogate data (Fig. 5a), revealing the rich dynamics of brain activity, which is further illustrated by the similarity matrices (Fig. 5b–d). The high correlation blocks off the diagonal line in the similarity matrix indicate recurrence of similar ReHo pattern, which only presented in real fMRI data.

Furthermore, to understand how ReHo in different ICNs may evolve with time, the spatial correlations of ReHo in different ICNs were also explored. The existence of recurrent ReHo patterns can be inferred from the similarity matrices (Supplementary Fig. S4). The results showed that ReHo in different ICNs fluctuated with considerable independence, indicated by their distinguishable recurrence patterns.

**Figure 6:**
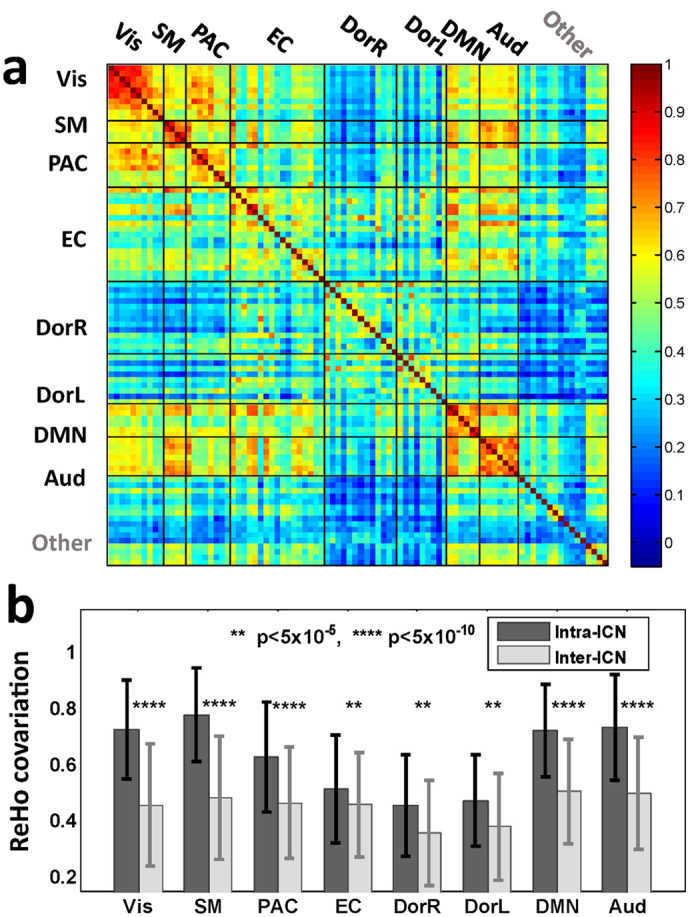
Temporal covariation of ReHo between ROIs. **(a)** ReHo covariation between ROIs defined by AAL template. Each entry in the matrix represents the covariation of ReHo between a pair of ROIs, as measured by Pearson’s correlation coefficient averaged across subjects. **(b)** Intra-ICN and inter-ICN ReHo covariation of all the ICNs. Error bars indicate standard deviation across subjects.

The dependency of ReHo fluctuation across different brain regions may contain information about local FC dynamics with respect to global brain organization. To explore this possibility, the 90 AAL ROIs were assigned to different ICNs according to maximum-overlap criterion. VisMed and VisLat were merged into one visual network for convenience, as VisLat only occupied two AAL ROIs. ROIs not belonging the above ICNs (e.g., hippocampi and subcortical nuclei) were categorized as ‘other’. Then the covariation of ReHo fluctuation between each pair of ROIs was measured by the Pearson’s correlation coefficient between the dynamic ReHo series of the two ROIs (Fig. 6a). This covariation clearly manifested a structured pattern, which was absent in surrogate data (Supplementary Fig. S5). We then defined the intra-ICN covariation as the ReHo covariation averaged across all ROI pairs within the same ICN, and the inter-ICN covariation as the average ReHo covariation between ROI pairs, with one ROI from the ICN under investigation, and the other ROI from the other ICNs. Paired t-test (N = 38) between intra-ICN covariation and inter-ICN covariation suggested that, all ICNs of interest exhibited significantly higher intra-ICN ReHo covariation over inter-ICN ReHo covariation (Fig. 6b). We also performed the same analysis using another template containing 268 ROIs (usually referred to as Shen268 template), in which all the ROIs are assigned to eight different functional networks^31^. Results showed that the ReHo covariation in this template also exhibited a clear structure, and the intra-network ReHo covariations were significantly higher than the corresponding inter-network covariations (Supplementary Fig. S6).

### Motor Task Modulates the Temporal Variability of ReHo

**Figure 7:**
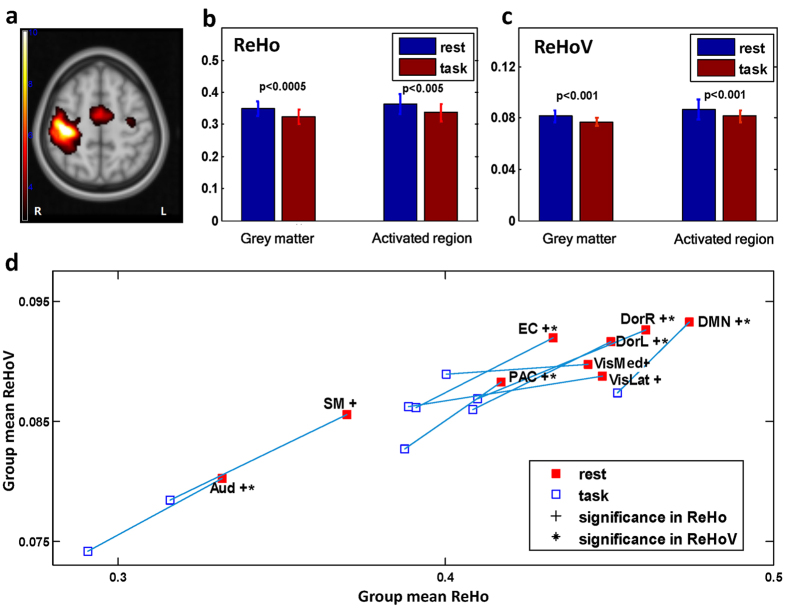
**(a)**Group-level activation map of HCO task (p < 0.01); **(b)** mean ReHo and **(c)** mean ReHoV in either whole grey matter or activated regions was significantly different between HCO task state and resting state; **(d)** group mean ReHo and mean ReHoV alterations induced by task for all the nine ICNs of interest, FDR corrected (q = 0.05).

The group-level activated regions of HCO task were shown in Fig. 7a (p < 0.01) and detailed in Supplementary Fig. S7. Voxel-wise paired t-test (N = 13) revealed significant decrease of ReHo in bilateral lingual gyri, right supramarginal gyrus, right superior frontal gyrus, and right middle frontal gyrus in the continuous HCO task comparing with resting state (false discovery rate (FDR) corrected, p < 0.0005, q = 0.05, see Supplementary Fig. S8), but no significant alteration in ReHoV appeared at voxel level after FDR correction. However, as illustrated in Fig. 7b,c, significant decreases of both mean ReHo and mean ReHoV during task performance could be observed in grey matter (defined as the total volume of all the 90 AAL ROIs) and, particularly, those task-activated regions. Furthermore, we performed an ICN-wise comparison with paired t-test. ReHoV significantly decreased in most of the ICNs except for the lateral visual network (VisLat), medial visual network (VisMed), and sensory-motor network (SM) (N = 13, FDR corrected, p = 0.013, q = 0.05), while ReHo significantly decreased in all ICNs (FDR corrected, p = 0.022, q = 0.05) (Fig. 7d). Note that this result was consistent across different length of sliding window (i.e., 40 sec, 60 sec, and 80 sec), and Fig. 7d presented the ReHoV result with sliding window of 60 sec.

## Discussion

In this study, we examined the dynamic fluctuation of local functional connectivity with ReHo algorithm. We found that ReHo variability positively correlates with ReHo, which cannot be reproduced by surrogate data. Furthermore, high ReHoV tended to appear in the global functional ‘hubs’ in the brain network, and stronger co-variation of ReHo existed between ROIs within the same ICNs than that between ROIs of difference ICNs. These two results suggested the association between ReHo fluctuation and brain function. Additionally, during an HCO task, ReHo variability was observed to be down-regulated in DMN, EC network, and visual networks, compared with resting-state. Our findings shed light on the intrinsic variability of local FC, and open up the possibility for further exploration of the dynamic properties of local FC.

High ReHo variability is located in high ReHo regions that were commonly regarded as local functional ‘hotspots’. The origin of such positive ReHo-ReHoV correlation was examined using surrogate test. While ReHoV and ReHo were also correlated in randomized data to some extent, the correlation was substantially weaker than that in the real fMRI data. This observation suggested that, in real human brain, the ReHo-ReHoV correlation may partly originate from the intrinsic feature of local FC network beyond the stochastic effect. In addition, we found a strong negative correlation between inter-regional FC and FCV (Supplementary Fig. S9), and FCV is especially prominent among ROI-pairs with weak FC. This result provides indirect evidence that the positive ReHo-ReHoV correlation was not simply a consequent result induced by statistic effect. Taken together, the local FC-FCV correlation is contributed by an unknown intrinsic source in the brain that remains to be explored.

Our results further suggested the functional relevance of ReHo fluctuation. Firstly, we found that DMN showed strongest ReHo level, which is consistent with previous findings^16^,^17^, suggesting that DMN serves as an important hub in the brain. Such functional centrality also appeared in global aspect: high ReHo ROIs, such as those in DMN and the visual networks, have more functional connections with distant brain regions. What’s more, both regional and global centrality were discovered to be accompanied by high ReHoV. The coexistence of local FC variability and global FC centrality in the hub regions could presumably be a result of multiple functional engagements. Such hypothesis could be supported by a recent MEG-based study^32^, which showed that DMN interacts with other networks when DMN itself is in a transient epoch of high internal correlation. In other words, the variability of within-network (or local) connectivity is closely linked to DMN’s functional centrality in the entire brain network. Although the BOLD signal is far more ‘sluggish’, and distinct from MEG signal with respect to the physiological processes they reveal, it is still possible that ReHoV is related to such global functional re-assembling. Therefore, we speculate that high degree of ReHoV in DMN is crucial for DMN’s role as a functional hub of brain.

The dynamic local FC reveals the rich and complex activity in human brain, which may not be adequately unveiled by global dynamic FC methods. While global dynamic FC researches often employ clustering techniques to extract different ‘modes’ of dynamic FC under the assumption that brain as an entirety evolves as a multi-stable system^3^,^4^, our preliminary observation on the temporal evolution of ReHo in ICNs suggested that local FC in different ICNs may exhibit relatively independent temporal dynamics. On one hand, each ICN could possibly display its own ‘multi-stability’ with respect to local FC dynamics, which is beyond the main focus of this study but worth further exploration. On the other hand, the dependency of ReHo fluctuation between different brain regions offered additional knowledge about the dynamics of ReHo in a global perspective, as was revealed by our analysis on the ReHo covariation across different brain regions. Intuitively, the pattern of covariation exhibited a clear structure that was unlikely to be the result of random temporal perturbation of ReHo, which reinforced our previous extrapolation on the functional significance of ReHo fluctuation. We then observed that the covariation was stronger for ROI pairs within the same ICNs. Furthermore, the pattern of ReHo covariation was also coherent with functional networks as defined by the Shen268 template. These findings suggested that the dynamics of regional FC reflects the global functional layout of the brain. Besides, the ReHo covariation matrix resembles the FC matrix, which is confirmed by an additional analysis on the relationship between FC and ReHo covariation, suggesting a strong correlation between them. In other words, strong FC between a pair of ROI suggests a strong ReHo covariation between this ROI pair, and vice versa (Supplementary Fig. S10). Since BOLD signal and ReHo reflect different neuro-physiological phenomena that happen in different temporal scales, the above observation turned out to be remarkable as it demonstrates an interplay between global FC and local FC dynamics. In other words, this phenomenon suggested that the brain can be conceived as a giant ‘network of networks’, composed of relatively independent but well-coordinated sub-systems, whose coordination, previously described in terms of FC, is now concordantly depicted by ReHo covariation across ROIs.

In this study, we also observed that ReHoV could be down-regulated by task, which was not limited to those brain regions directly involved in the task. This observation is concordant with a recent study on global FCV^5^, in which FCVs across and within various sub-networks were reported to be significantly lower during a global-local selective attention task paradigm than in resting state. Additionally, the FCV decrease was dependent on the increase of task difficulty, suggesting that a decrease in FC variability is crucial for task performance. On the other hand, higher FCV could be related to the unconstrained mind-set in resting state: an interesting study has shown that FCV in DMN positively correlates with the degree of ongoing daydreaming^7^. As rs-fMRI is unconstrained with respect to internal cognition, resembling daydreaming to some degree, the decreased FCV and ReHoV in task performance may be related to the reduced shifting frequency of brain’s ‘transient modes’ due to the presence of task demand. By using co-activation pattern (CAP) method to represent the ‘transient modes’, Chen *et al.* demonstrated that both DMN associated transient modes (i.e., CAPs) and EC associated transient modes were influenced by task performing^6^. In sustained working memory task, the number of temporally dominant transient modes (i.e., the CAPs that showed up most frequently) decreased compared with resting state, and the temporal fraction of the first dominant transient mode was higher, indicating that brain becomes more involved in a specific ‘mode’ during a task, thus becoming more ‘stable’. Taken together, our discovery further supports the hypothesis that FCV can be attributed to the daydreaming-like, unconstrained mind process in the resting state brain, which is suppressed in task performance. More importantly, our finding indicates that the modulatory effect of task on the variability of FC also exists in a regional, sub-centimetre scale. Nevertheless, our observation is based on a specific task performed by a small group of subjects, therefore, further studies are desired in order to draw a clearer picture on the task modulation on ReHoV.

The statistical analysis of the HCO continuous task dataset is one major issue to be discussed. From a voxel-wise perspective, task modulation appeared to be more evident in ReHo than in ReHoV. However, the paired t-test of the average ReHoV over whole grey matter revealed that ReHoV was also significantly modulated by task, suggesting that the alteration of ReHoV was concealed by noise and was spatially diffuse (as noise can be suppressed by the averaging process, and spatially prevalent phenomenon is less susceptible to partial volume effect). In order to address the spatial description in this phenomenon and avoid the overly strict voxel-wise FDR correction, ICN-wise average was used as a reconciliation between whole-brain comparison and voxel-wise comparison, which revealed significant alteration of ReHoV after FDR correction. Nevertheless, this approach is still biased against the discovery of significant regions whose volumes are considerably smaller than the ICNs they belong to. One possible remedy to this issue is to use a dataset with larger sample size and longer scan time, which yields higher statistical power. Another methodological concern is the 3-dimensional algorithm we used to compute ReHo. As cortical surface has complex folding patterns (sulcus and gyrus), the neighbouring voxels defined in 3d ReHo algorithm may not be the real neighbours in geodesic distance^27^, introducing some confounding effects. Therefore, in future studies, it could be beneficial to explore ReHo and its dynamics using surface-based 2d-ReHo algorithm^27^,^33^.

In this study, the primary goal was to explore the dynamic fluctuation of ReHo. Apparently, more effort is needed to explore the nature of local FC variability. Many open questions remain. For example, does the ReHoV patterns alter or deviate in development or pathological situations? Will the dynamics of ReHo reveal similar multi-stability of brain, as revealed by global FC dynamics? What is the mechanism that drives the covariation pattern of ReHo dynamics into a familiar functional structure depicted by the global FC? Answers to these questions could offer more insights into the theory and potential applications of dynamic ReHo analysis.

In conclusion, we found that strong local connectivity of the brain, measured by ReHo, also manifested strong temporal fluctuation. The strong positive correlation between ReHoV and ReHo was independent of algorithm, window size, and the definition of fluctuation, and cannot be fully reproduced in randomized surrogate data, implying that this relationship between ReHoV and ReHo should be an intrinsic feature of brain rather than a simple side-effect of regional connectivity algorithm. Two observation associated the dynamic ReHo fluctuation with brain’s functional layout: (i) regions with high ReHoV tended to be functional ‘hubs’ connecting distant brain regions, probably due to the functional modulations from multiple distant regions that connected to these ‘hubs’; (ii) the intra-ICN ReHo covariation was greater than inter-ICN ReHo covariation. In addition, during continuous HCO task, ReHo variability was observed to be down-regulated, which supported the hypothesis that FC variability is partly caused by daydreaming-like mind process, and indicated that task modulation on FC variability also exists on a regional scale. In conclusion, our study introduces the prospect of performing dynamic analysis of regional brain connectivity with ReHo.

## Materials and Methods

### Human Connectome Project Data

This dataset were obtained from the WU-Minn Human Connectome Project^34^ database (http://humanconnectome.org/). We chose the ‘40-unrelated subject’ group included in the ‘500 Subjects MR + MEG2 Release’. Two subjects were excluded due to the anomalies in brain structure. The data for the remaining 17 males and 21 females were used for further analysis. Each subject underwent two rs-fMRI sessions, each consisting of two runs with different encoding directions. Here we used the rs-fMRI data in session 2 with left-to-right phase encoding, assuming that subjects would be better adapted to the scanner in the second session, and possibly less affected by the environment in the scanner.

Subjects were scanned on a customized Siemens (Erlangen, Germany) 3 T ‘Connectome Skyra’ at Washington University in St. Louis. T1 images were acquired using 3D MPRAGE sequence, TR = 2400 ms, TE = 2.14 ms, TI = 1000 ms, flip angle = 8 deg, FOV = 224 × 224 mm, voxel size = 0.7 mm isotropic. Rs-fMRI data was acquired using gradient-echo EPI sequence, with TR = 720 ms, TE = 33.1 ms, flip angle = 52 deg, FOV = 208 × 180 mm, multiband factor^35^ = 8, echo spacing = 0.58 ms, band width = 2290 Hz/Px, 72 slices of 2 mm isotropic voxels. Each run of rs-fMRI scan lasted for 14 min 33 sec, producing 1200 frames of 3D images. In the scan, subjects were required to open their eyes, relax, and fixate on a projected bright cross-hair on a dark background. Detailed scanning protocol has been described in^36^.

We adopted the minimally pre-processed fMRI data in this study. This pre-processing pipeline^37^ incorporated functions in FSL^38^,^39^ and FreeSurfer^40^, including gradient distortion correction, motion correction, field map pre-processing, distortion correction, EPI to T1 registration, resampling original EPI frames to atlas space, intensity normalization, and ribbon-based native-to-standard space registration. Slice-timing correction and temporal filtering were not implemented in the pipeline.

Additional pre-processing was performed as follows. The data was de-trended to remove scanner drift. Then, the first 4 volumes of the data were discarded to exclude unstable signal at the beginning of the scan. To reduce the cost of computation, we down-sampled the BOLD signal by a factor of 2. A band-pass filter (0.01 to 0.1 Hz) was then applied to the data. Motion parameters were used to regress out motion-related nuisance^41^. No additional spatial smoothing was performed as smoothing introduces significant bias in the following ReHo computation. We did not perform slice-timing correction to the data, as the TR of rs-fMRI data was considerably short (0.72 second) and the effect of time lags between slices was limited.

### Resting-State and Continuous Motor Task Data

An additional dataset was used to investigate how task might modulate ReHoV. Data acquisition was performed at Rui Jin Hospital affiliated to the School of Medicine of Shanghai Jiao Tong University, Shanghai, China. The experiment was in accordance with the sixth revision of the Declaration of Helsinki, and the protocols were reviewed and approved by the Ethics Committee of Shanghai Second People’s Hospital, Shanghai, China. All subjects were provided with a written informed consent. Thirteen middle-aged, right-handed subjects (age = 53 ± 6.52, 7 males) without psychiatric or neurological disorder were recruited. Subjects were scanned on a 3 T GE Signa Excite Gemse MR scanner (GE Healthcare, Milwaukee, WI, USA). Structural images were obtained using a sagittal 3D T1-MPRAGE sequence (TR = 5.824 ms, TE = 1.784 ms, TI = 450 ms, FOV = 256 × 256 mm, voxel size = 1 mm isotropic, flip angle = 12 deg) with 196 contiguous sagittal slices (1 mm thick) covering the whole brain. fMRI data were collected with scanning parameters: TR = 2000 ms, TE = 30 ms, voxel size = 3.75 × 3.75 mm, slice thickness = 4 mm.

There were three fMRI sessions in the experiment. In the resting session, subjects were told to remain motionless, relaxed, and awake for 6 minutes. During the continuous task session, subjects were instructed to perform HCO task with their left hand at a rate of once per second (paced by cues displayed on the screen) for 4 minutes and 20 seconds. The third session, a block-design task, consisted of six resting blocks alternated with five 20-second HCO task blocks. All of the three sessions were preceded by an 8-second preparing period. The whole experiment procedures were monitored by a physician to make sure that the subjects performed the experiment correctly. ReHo and ReHoV were calculated for rs-fMRI data and continuous HCO fMRI data. The block-design fMRI data were used to locate task-related regions.

The rs-fMRI data and the HCO fMRI data went through identical pre-processing steps as follows using DPARSF toolbox^41^. The first 4 frames were removed, and raw images were corrected in slice-timing. Then, all slices were realigned and normalized by DARTEL^42^ algorithm, and band-pass (0.01–0.1 Hz) filtered. The nuisance signals (motion, white matted signal, CSF signal, and whole brain mean signal) were then regressed out. Finally, in order to allow comparison between rs-fMRI data and HCO fMRI data, the same length of rs-fMRI and HCO data were selected (i.e., 126 volumes).

### ReHo and ReHoV Computation

ReHo measures voxel-wise short-distance FC with Kendall’s coefficient of concordance^14^:


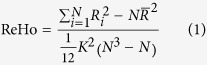


where *N* denotes the length of the time series, *K* = 27 is the size of the voxel cluster containing 3 × 3 × 3 adjacent voxels, *R*_*i*_ denotes the summation of the rankings of BOLD signal amplitude of all *K* voxels at the *i*th time point, and 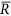 is the mean of *R*_*i*_. The ReHo algorithm was implemented in Matlab 2013a (Mathworks Inc., Natick, Massachusetts, USA) with codes adopted from the REST Toolbox^43^. To explore whether the observed phenomenon was specific to ReHo algorithm, we used ILC as an alternative algorithm, which is defined as,


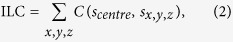


where *s* is BOLD signal, *C(S*_*centre*_ *, S*_*x,y,z*_) denotes the Pearson’s correlation between the BOLD signal of centre voxel (i.e., *S*_*centre*_) and the BOLD signal of a neighbouring voxel (i.e., *S*_*x,y,z*_), *x, y* and *z* denote the location of neighbouring voxels around the centre voxel, and the neighbourhood was also defined as a 3 × 3 × 3 cluster of voxels.

We first calculated the ReHo using the data from the entire scan. To calculate ReHoV for the Human Connectome Project data, scans were split into sliding time-windows, with each lasting 160 TRs (i.e., 1 min 55.2 sec) and 75% overlap between two successive windows. Then, ReHoV was defined as the standard deviation of ReHo across windows for each voxel. To confirm our results regarding ReHoV, we also tested different window lengths and overlaps.

For the resting-state and continuous HCO task data, ReHoV was computed using sliding windows with 30 TR length (i.e., 60 sec) and 10 TR increment, as longer sliding window length will yield fewer number of windows, and hence reduce the accuracy of ReHoV estimation. To ensure the consistency of our finding, we also analysed ReHoV with window length of 20 TR and 40 TR respectively.

Note that, considering the different duration (approx. 15 min for the first dataset and 4 min 20 sec for the second dataset) and TR (0.72 sec for the first dataset and 2 sec for the second dataset) in these two dataset, ReHoV was calculated with different window lengths in these two dataset. For both datasets, we also applied several different window length, which were common in many studies^4^,^10^,^12^,^44^,^45^, so as to test the consistency of our observation.

### Generating Surrogate Datasets

To determine whether the correlation between ReHo and ReHoV is inherent, a comparison between real fMRI data and randomized surrogate data was carried out. Surrogate dataset was generated by shuffling the phase of the real pre-processed fMRI time series for each in the frequency domain^4^,^46^. In this way, the mean and the variance of the BOLD signal are preserved, but the temporal correlation between a voxel and its neighbours no longer exists.

Note that fMRI images possess an intrinsic smoothness (i.e., time series from two adjacent voxels will generally be more similar comparing to non-adjacent voxel pairs, even in the absence of Gaussian smoothing). Such intrinsic smoothness contributes to the ReHo level, since spatial smoothness enhances the temporal similarity of time courses of neighbouring voxels. However, phase-randomization is expected to roughen the image significantly. This “side-effect” is not desirable because we wanted to obtain artificial data that is as similar to original data as possible. To remedy this “side-effect”, the phase-randomized dataset was smoothed such that the surrogate dataset possesses the spatial smoothness similar to the real fMRI data. The smoothness, or spatial autocorrelation structure, can be modelled as a result of a convolution of real spatial signal with a Gaussian filter with an unknown full-width-half-maximum (FWHM) window to be estimated^47^. The smoothness estimation was implemented by REST toolbox^43^. In our study, the smoothness of real fMRI image was estimated by taking the average of the smoothness estimations of three frames (one in the beginning, one in the end, and one in the middle of the scan). Then a Gaussian filter with the estimated FWHM window was used to smooth the phase-randomized data, frame by frame, yielding smoothed surrogate images possessing similar smoothness to the real fMRI data.

### Functional Module Definition by Independent Component Analysis

We used probabilistic ICA^48^ in FSL MELODIC^38^,^49^ to extract ICNs from the rs-fMRI data. Specifically, to explore common spatial patterns of all the ICNs across all subjects, group-ICA was performed, which concatenated the fMRI data of all the subjects temporally to make a single 4-D image, and then decomposed this image into independent components. After visual inspection, the independent components related to functional activities were selected as ICNs. Z-statistic maps of the selected ICNs were thresholded at 0.95 to make common binary masks for all subjects.

### Functional Connectivity and Graph Theoretical Analysis

The AAL template^50^ was applied to parcellate the brain into 90 ROIs. Each ROI was defined as a node in brain network. The mean time course in each ROI was extracted as its representative time course, and the Pearson’s correlation coefficient of the representative time series of two ROIs was calculated as functional connectivity between the two ROIs. For each subject, functional connectivity was computed for all possible pairs of the 90 ROIs, generating a 90 × 90 association matrix. Nodal strength, which represents the sum of the weights of all the edges connecting to a specific node (i.e., ROI defined by AAL template), was calculated for each node of each subject. Negatively weighted edges were excluded before calculating nodal strength in order to avoid misinterpreting negative edges^51^. Then the ReHo-nodal strength correlation and ReHoV-nodal strength correlation were examined to explore the functional roles of ReHo and ReHoV from the perspective of the brain network.

### Statistical Analysis

Statistical analysis was carried out using the Matlab statistic toolbox. Kolmogorov-Smirnov test was carried out before T-test and regression analysis to ensure the normality of the data before performing correlation analysis and t-test. Multiple comparisons were performed with false discovery rate (FDR) correction^52^.

Mean ReHo and mean ReHoV in the 90 ROIs defined by AAL template were extracted. Then the Pearson’s correlation was computed between mean ReHo and mean ReHoV across ROIs. To examine whether the result are dependent on ROI definition or not, we further adopted a finer-parcellated template called AAL1024 (see Supplementary Fig. S1a), which further divides 90 AAL ROIs into 1024 ROIs^53^,^54^.

In the surrogate test, we compared the ReHo-ReHoV similarity across the real dataset, phase-randomized dataset, and phase-randomized-and-smoothed dataset. The degree of similarity was measured by the spatial correlation between ReHo and ReHoV.

Activation analysis was performed to the block-design fMRI data to locate the task-related regions. For the first-level analysis of each subject, general linear model (GLM) in SPM8 was used to generate the individual activation map. The box-car vectors for HCO task blocks were convolved with hemodynamic response function and the head movement parameters were included as covariates to remove the artefacts induced by head motions. After that, the one-sample t-test based on single-subject contrasts obtained in the first-level analysis was performed for all subjects, yielding group-level activation map (p < 0.01).

## Additional Information

**How to cite this article**: Deng, L. *et al.* Characterizing dynamic local functional connectivity in the human brain. *Sci. Rep.* **6**, 26976; doi: 10.1038/srep26976 (2016).

## Supplementary Material

Supplementary Information
