# Supplementary material for: Characterizing dynamic local functional connectivity in the human brain
Source: Sci Rep. 2016 May 27;6:26976. doi: 10.1038/srep26976 (PMC4882585; doi:10.1038/srep26976)
Supplement: Supplementary Information [file srep26976-s1.doc]

**Characterizing dynamic local functional connectivity in human brain**

Lifu Deng, Junfeng Sun^*^, Lin Cheng, Shanbao Tong

**Supplementary Information**

**Supplementary Figure S1**

**(a)** Scatter plot of ROI-wise mean ReHo and mean ReHoV in ROIs defined by AAL1024 template; **(b)** scatter plot of ROI-wise mean ReHo and mean ReHoV in ROIs defined by AAL90 template, in which ReHoV was calculated using three different window lengths (80-frame, 60-frame, and 40-frame) and three different proportions of window overlap (75%, 50%, and 25%). Shorter windows yielded larger variability as expected, while window overlap did not affect ReHoV much.


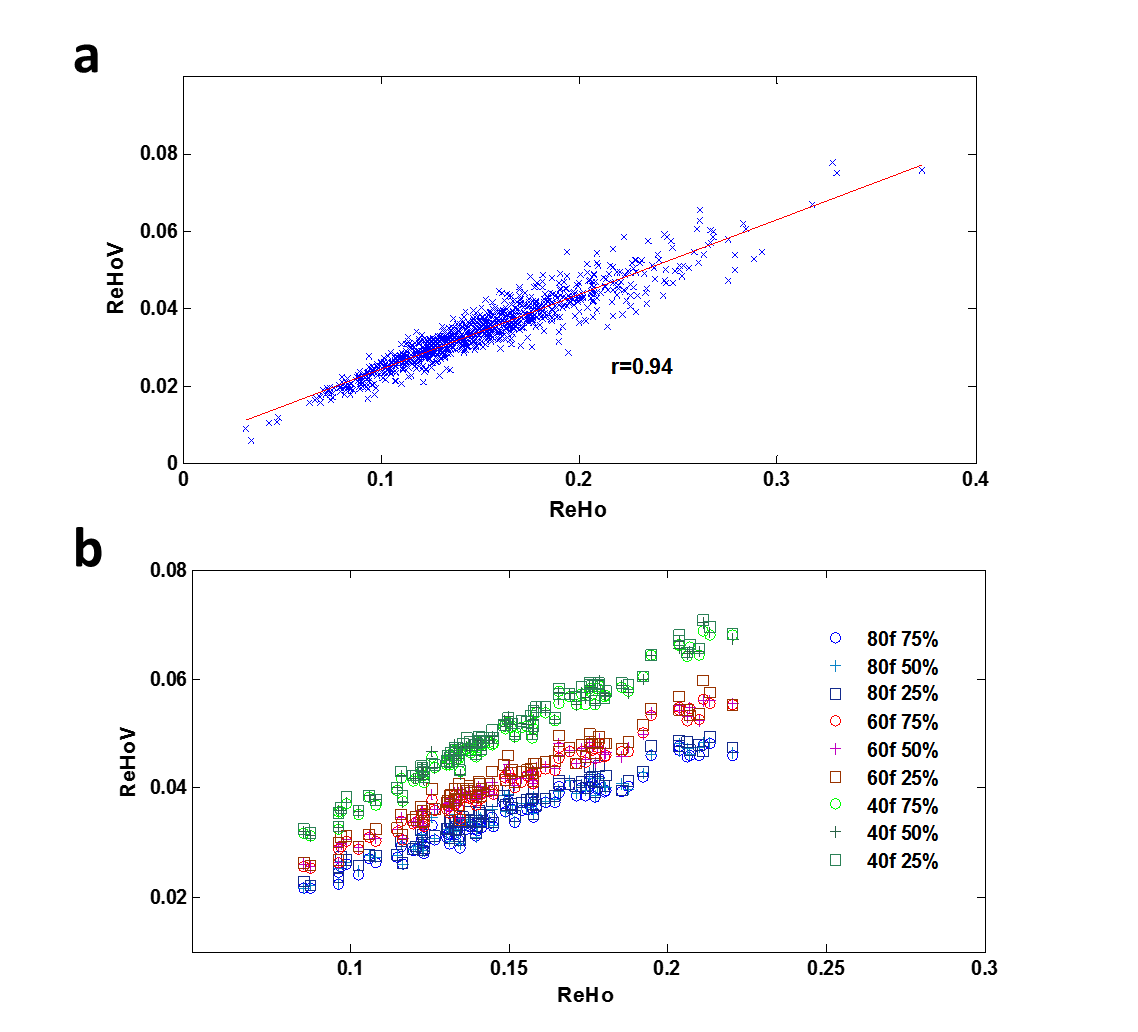


**Supplementary Table S2**

Summary of ReHo-ReHoV correlation among subjects in Human Connectome Project data

|  | Pearson’s correlation (mean ± std) | Range of Pearson’s correlation |
| --- | --- | --- |
| AAL90 | 0.953±0.023 | [0.889, 0.983] |
| AAL1024 | 0.922±0.020 | [0.860, 0.961] |

**Supplementary Table S3**

Summary of individual ReHo-nodal strength correlation and ReHoV-nodal strength correlation

|  | Number of subjects having significant correlation | Subjects showing no significant correlation  and the corresponding p-value |
| --- | --- | --- |
| ReHo –  Nodal strength | 33 | ID=113619, p=0.145; ID=130013, p=0.193;  ID=149337, p=0.963; ID=149539, p=0.295;  ID=397760, p=0.721 |
| ReHoV – Nodal strength | 31 | ID=111312, p=0.105; ID=113619, p=0.095;  ID=130013, p=0.808; ID=149337, p=0.774;  ID=149539, p=0.346; ID=156637, p=0.120;  ID=397760, p=0.386 |

**Supplementary Figure S4**

Similarity matrices showing the spatial correlation between ReHo maps of any pair of windows in nine ICNs respectively. The data presented here is from a typical subject. The numbers of x-axis and y-axis denote the window indexes.


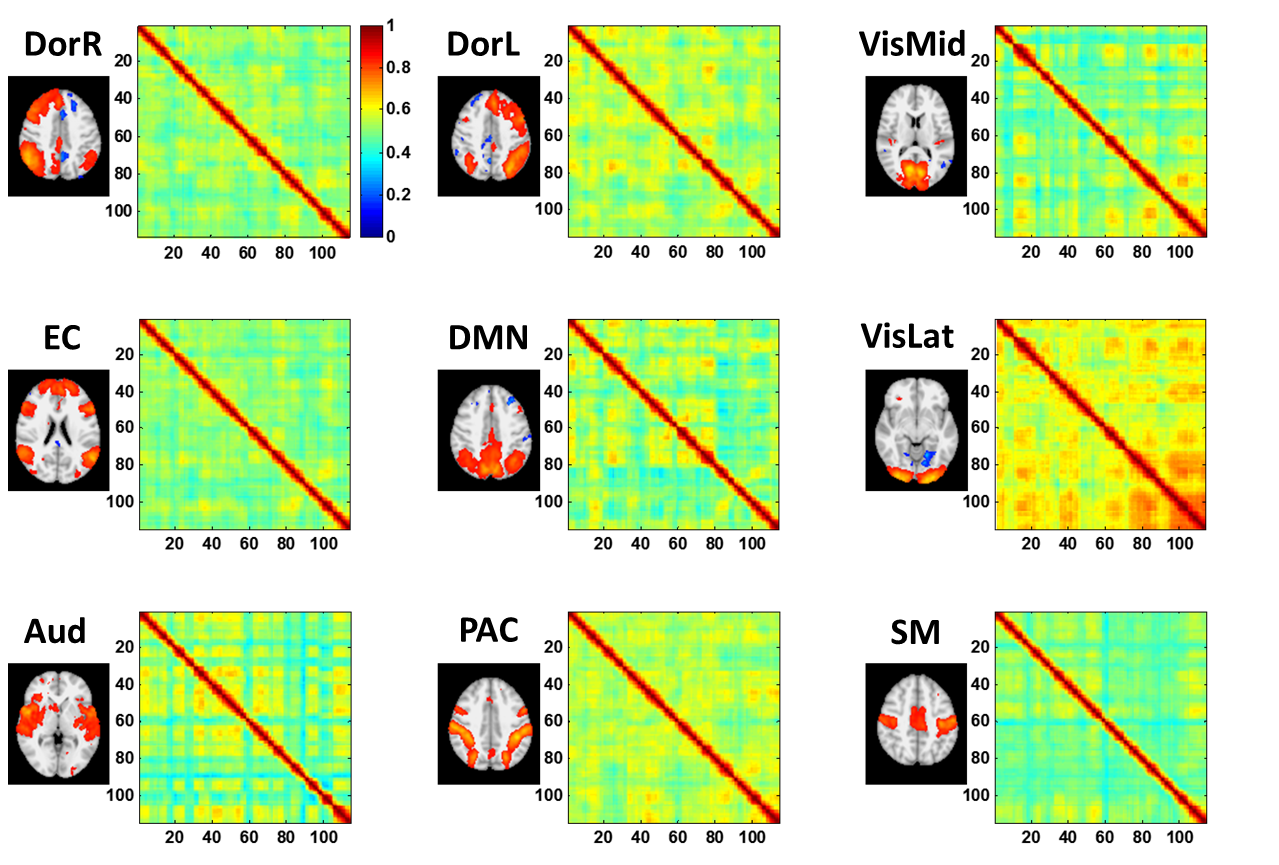


**Supplementary Figure S5**

The ReHo covariation matrix of one typical subject, and the ReHo covariation matrix from the surrogate data of this subject.


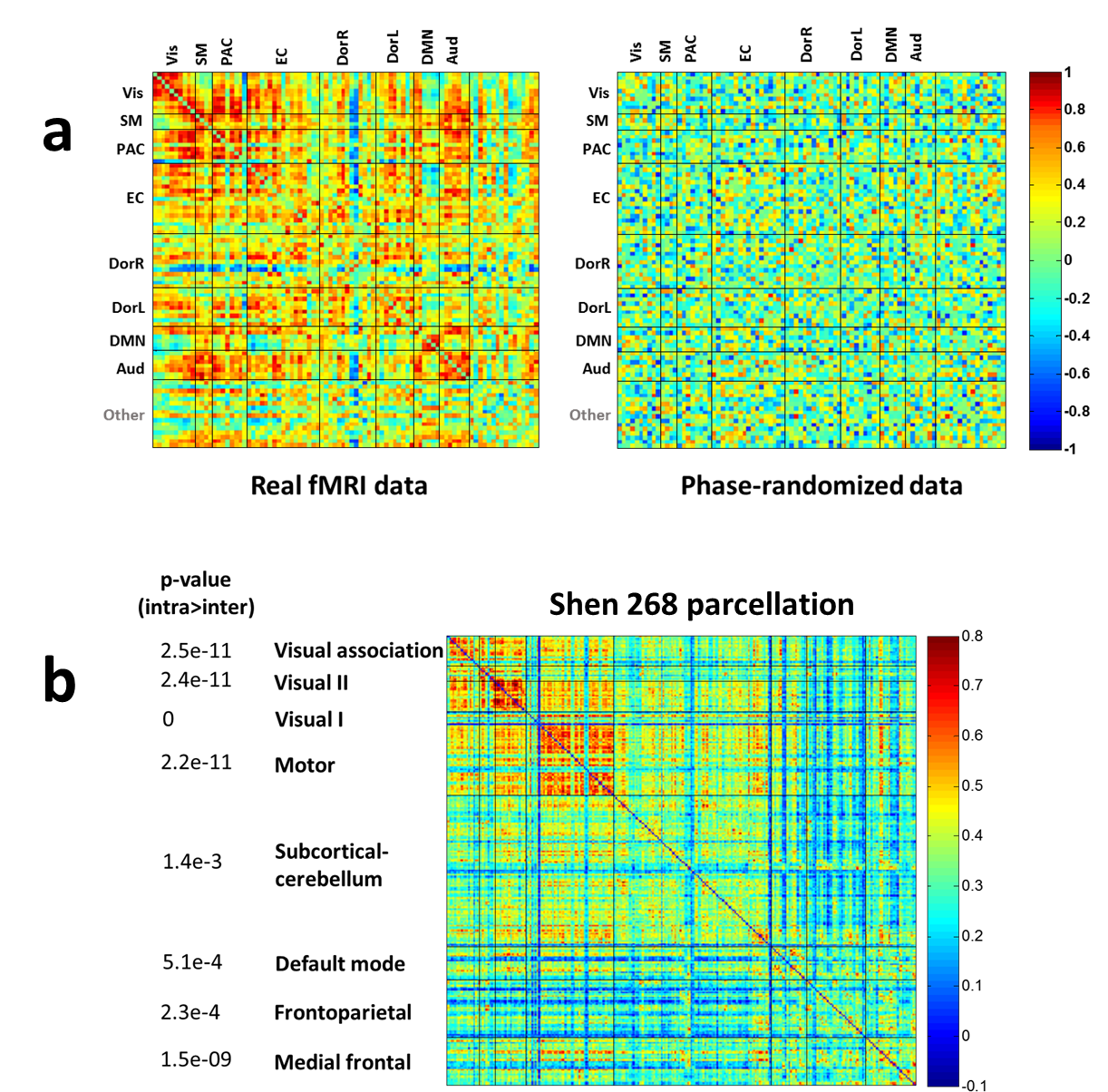


**Supplementary Figure S6**

**(a)** The group-averaged ReHo covariation matrix derived from Shen268 template. **(b)** Intra-network and inter-network ReHo covariation of all the functional networks. Error bars indicate standard deviation across subjects. All functional networks exhibited significantly higher intra-network ReHo covariation (paired t-test, p<0.005).

**
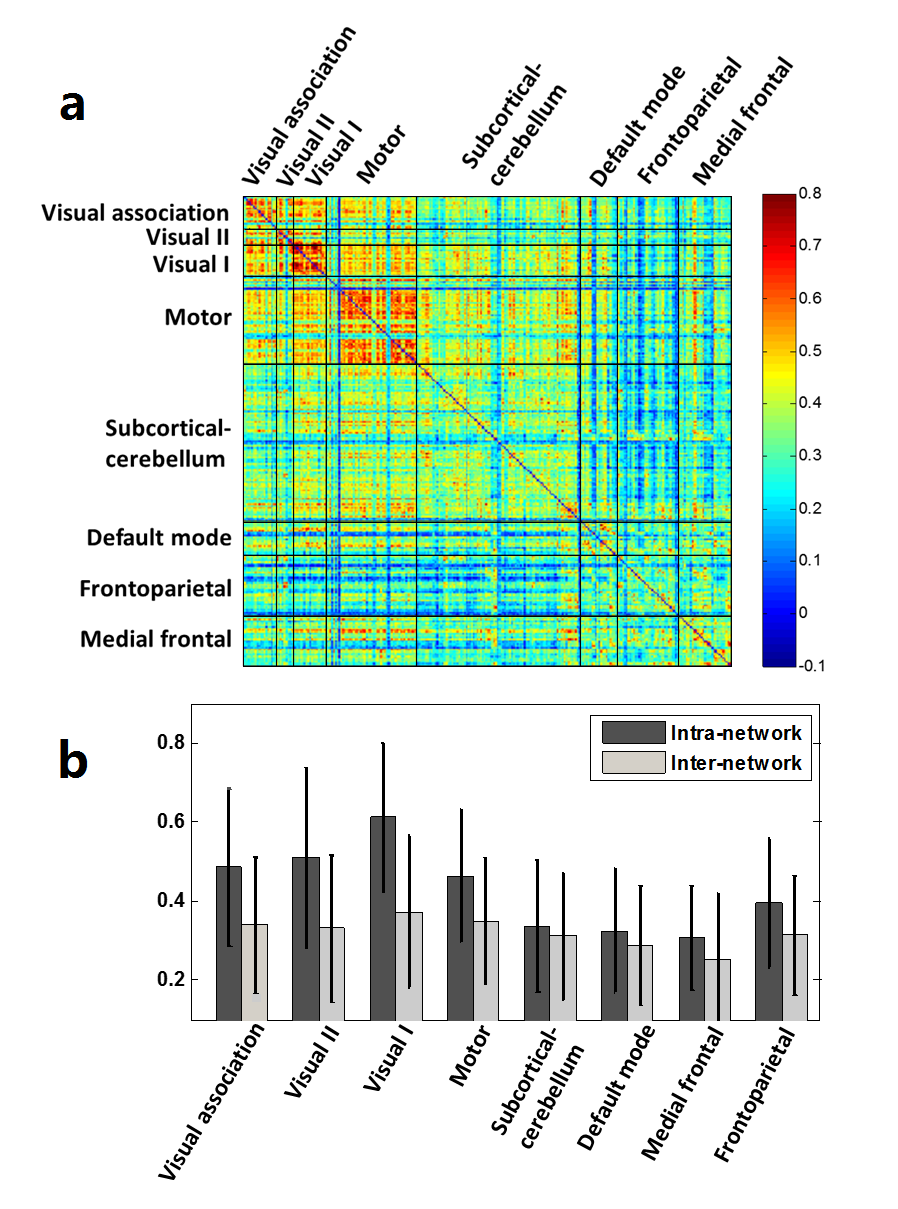
**

**Supplementary Figure S7**

HCO task activated regions (p<0.01), and the MNI coordinates of the peak voxels


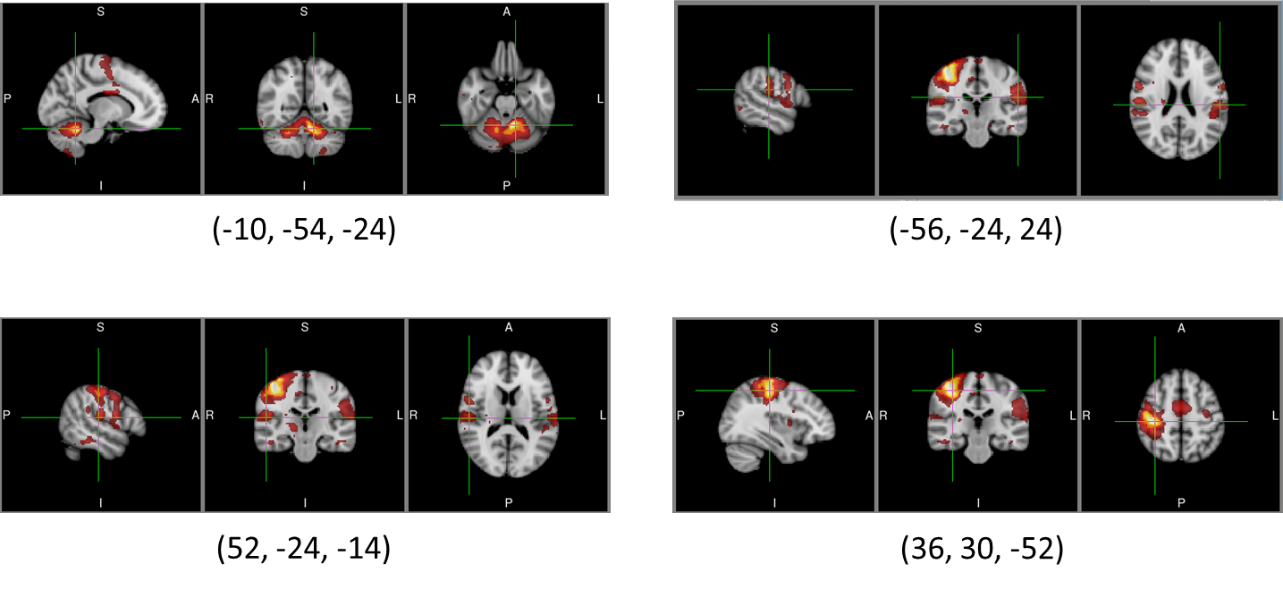


**Supplementary Figure S8**

Regions showing significant decrease of ReHo in HCO task compared with resting state (p<0.0005, FDR corrected, α=0.05).


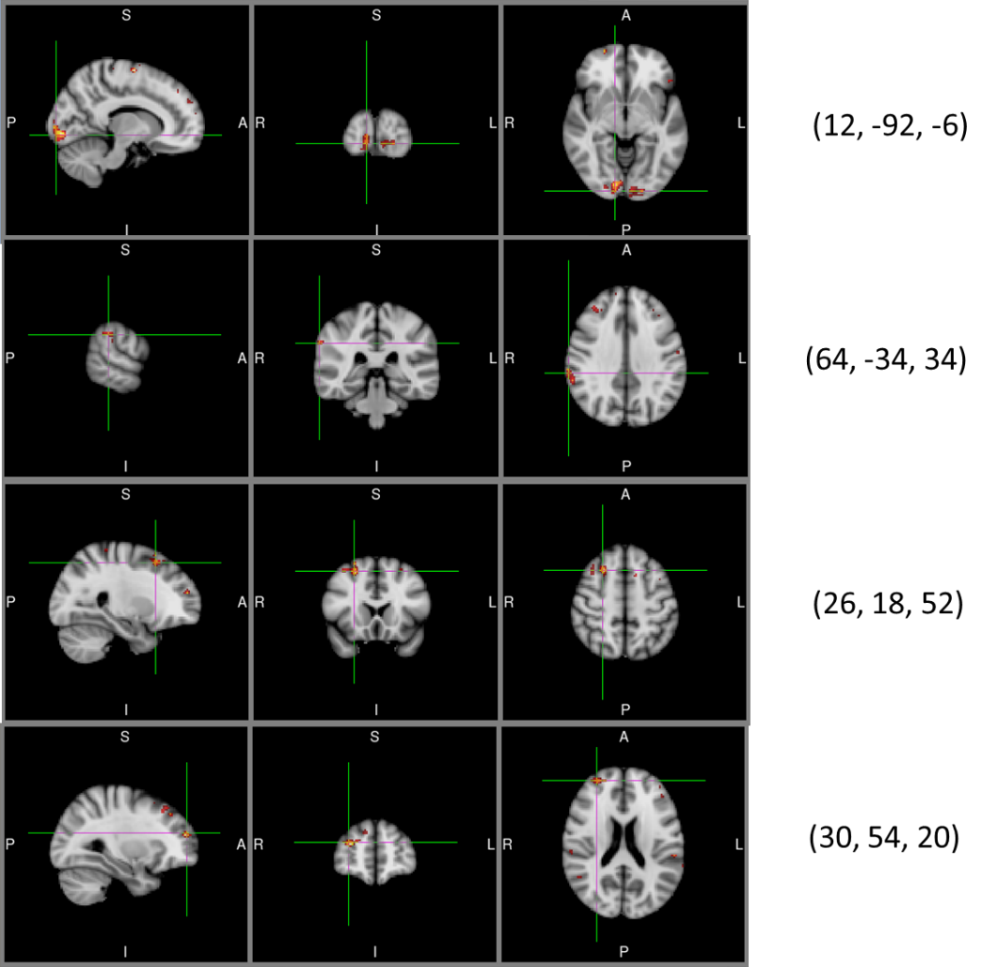


**Supplementary Figure S9**

The negative FC – FCV correlation from a typical subject. Each dot represents the FC and FCV between a pair of ROIs which were defined by AAL90 template. FC is defined as the Pearson’s correlation coefficient between the two representative BOLD time courses of the two ROIs, while FCV is defined as the standard deviation of FC series obtained in sliding windows. Each sliding window lasted 160 TRs, and has a 75% overlap with adjacent windows.

**Supplementary Figure S10**

Group average ReHo covariation positively correlates with group average FC. Each dot stands for the ReHo covariation and FC between a pair of ROIs.
